# The Dynamic of the Splicing of bZIP60 and the Proteins Encoded by the Spliced and Unspliced mRNAs Reveals Some Unique Features during the Activation of UPR in *Arabidopsis thaliana*

**DOI:** 10.1371/journal.pone.0122936

**Published:** 2015-04-10

**Authors:** Juan Parra-Rojas, Adrian A. Moreno, Irina Mitina, Ariel Orellana

**Affiliations:** Centro de Biotecnología Vegetal, FONDAP Center for Genome Regulation, Facultad de Ciencias Biológicas, Universidad Andrés Bello, Santiago, Chile; National University of Rosario, ARGENTINA

## Abstract

The unfolded protein response (UPR) is a signaling pathway that is activated when the workload of the endoplasmic reticulum (ER) is surpassed. IRE1 is a sensor involved in triggering the UPR and plays a key role in the unconventional splicing of an mRNA leading to the formation of a transcription factor that up-regulates the transcription of genes that play a role in restoring the homeostasis in the ER. In plants, bZIP60 is the substrate for IRE1; however, questions such as what is the dynamics of the splicing of bZIP60 and the fate of the proteins encoded by the spliced and unspliced forms of the mRNA, remain unanswered. In the present work, we analyzed the processing of bZIP60 by determining the levels of the spliced form mRNA in plants exposed to different conditions that trigger UPR. The results show that induction of ER stress increases the content of the spliced form of bZIP60 (bZIP60s) reaching a maximum, that depending on the stimuli, varied between 30 min or 2 hrs. In most cases, this was followed by a decrease in the content. In contrast to other eukaryotes, the splicing never occurred to full extent. The content of bZIP60s changed among different organs upon induction of the UPR suggesting that splicing is regulated differentially throughout the plant. In addition, we analyzed the distribution of a GFP-tagged version of bZIP60 when UPR was activated. A good correlation between splicing of bZIP60 and localization of the protein in the nucleus was observed. No fluorescence was observed under basal conditions, but interestingly, the fluorescence was recovered and found to co-localize with an ER marker upon treatment with an inhibitor of the proteasome. Our results indicate that the dynamics of bZIP60, both the mRNA and the protein, are highly dynamic processes which are tissue-specific and stimulus-dependent.

## Introduction

The unfolded protein response (UPR) is an important cellular mechanism that controls the homeostasis in the endoplasmic reticulum (ER). This mechanism is based on sensing the accumulation of unfolded proteins at the ER lumen by several sensors located at the ER membrane [1]. Upon their activation, a signaling pathway leading to the transcription of genes involved in the synthesis, folding and degradation of proteins at the ER, is activated. One of the most studied and conserved sensors across eukaryotes is the inositol-requiring enzyme 1 (IRE1) [2,3]. IRE1 is a transmembrane protein that has a luminal domain capable to sense the accumulation of unfolded proteins trough direct interaction with them or by an indirect manner that depends on the availability of the binding protein chaperone (BiP) [4,5,6,7,8]. It has been shown that upon its activation IRE1 can process the mRNA of a transcription factor, which is then re-ligated through the action of a tRNA ligase [9,10,11]. This phenomenon, known as an unconventional splicing, has been described in different organisms such as yeast, insects, mammals and more recently in plants [12,13,14,15]. In *Arabidopsis thaliana* it has been described that the mRNA corresponding to bZIP60 can be processed by IRE1 during the unfolded protein response triggered by chemicals that induce the accumulation of unfolded proteins [15,16,17]. The processing is abolished on IRE1 mutant plants, thus establishing a link between the activation of IRE1 and the splicing of bZIP60 [15,16,17].

In plants, it has been described that several abiotic and biotic stresses can trigger the IRE1 signaling pathway, leading to the splicing of bZIP60 [15,17,18]. However, our current knowledge about how the processing of bZIP60 takes place during different stresses is limited. Recent reports indicated that processing of bZIP60 could be sustained at least ten hours under salicylic acid treatment [19]. In contrast, in other eukaryotes, it has been described that the processing of orthologs of bZIP60 such as HAC1 in yeast or XBP1 in mammals should be attenuated to support cell viability even if the stimulus that triggers UPR is still present [20,21,22]. In addition, the fact that plants are sessile organisms suggests that activation of UPR should be an intermittent process during the plant life cycle. For example, plants have to respond to higher temperatures during the day than in the night; therefore, it is likely that activation of UPR may be regulated differentially during day and night.

Upon the formation of the spliced form of bZIP60 mRNA, the protein is translated and then migrates to the nucleus. Support for this hypothesis has been provided by Iwata et al. [23], where *Arabidopsis thaliana* suspension cells incubated with tunicamycin (Tm) accumulated bZIP60s in the nuclear fraction, whereas the protein derived from the unspliced form was present in the total fraction but not in the nucleus. In addition, Deng et al. [15], showed that bZIP60s is found in the nucleus when the spliced form of the bZIP60 mRNA is directly expressed in BY-2 cells. Finally, Nagashima et al. [16] found that in seedlings treated with DTT and Tm, the majority of the protein corresponded to the product encoded by the spliced form of bZIP60. Intriguingly, neither the proteins encoded by bZIP60u nor bZIP60s were detected in basal conditions, despite the presence of the bZIP60 mRNA. Even though these results support the idea that bZIP60s is translocated to the nucleus when UPR is activated, this poses a question regarding the dynamic of the proteins derived from bZIP60 in basal conditions and during the activation of UPR.

In the present work, we analyzed the processing of bZIP60 by determining the levels of the spliced form of the mRNA in plants exposed, during several hours and in a reiterative manner, to conditions that trigger UPR. In addition, we analyzed the cellular distribution of the bZIP60 protein when UPR was activated *in planta* by using a transgenic line expressing the green fluorescent protein (GFP) fused to bZIP60 under the control of its endogenous promoter. The results indicate that the induction of stress in the ER promotes the splicing of bZIP60 continuously, reaching a maximum between 0.5–2 hrs followed by a decrease in the content of bZIP60s. Despite this, re-applications of the stimuli induce the splicing again. In contrast to what is observed in some eukaryotes [20,21,22], the splicing of the mRNA never occurs to full extent, since the unspliced form is always present in higher amounts than the spliced form, suggesting that only a fraction of the total bZIP60 mRNA undergoes this processing. Furthermore, the splicing of bZIP60 correlates with the accumulation of GFP-bZIP60 in the nucleus. Intriguingly, no fluorescence was observed under basal condition, despite the use of the endogenous promoter of the gene, suggesting that the protein derived from the unspliced form of bZIP60 is unstable. Interestingly, the fluorescence of GFP-tagged bZIP60u protein was recovered and found that co-localizes with an ER-marker upon treatment with an inhibitor of the proteasome. Our results indicate that the dynamics of bZIP60, both at the mRNA and protein levels, are highly dynamic processes which are tissue-specific and stimulus-dependent.

## Materials and Methods

### Plant material and general growth conditions

In the present work we used Arabidopsis (*Arabidopsis thaliana*) wild type plants ecotype Columbia-0 (Col-0) and the insertion mutant lines: *ire1a ire1b* mutant [17], *bzip28* (SALK_123659) [24], *bzip17* (SALK_104326) [25] and *bzip60* (SAIL_283_B03) [15]. Seeds were stratified at 4°C for 2 days and grown in soil or MS media as described for each experiment. All the plants were grown at 21°C under long day conditions (16 h light/8 h dark) employing white light (~63 μE m2 s-1).

### Transgenic line used in this study

For the construction of the transgenic plants expressing GFP-tagged bZIP60 under the control of its own promoter, a 1.2 kB promoter region of bZIP60 [26] was amplified using primers 5’-ACA AGT TCG GTT GAT AAG CAA GTC CAA AA-3’ and 5’-GGT CAA AAA AAA AAA AAT ATA CAA AGA AGA AAA AAA AAA GCG-3’ and subcloned in pENTR-TOPO 5’ vector (Invitrogen, Life Technologies). The genomic region of bZIP60 was amplified using primers 5’-GGGG ACAGCTTTCTTGTACAAAGTGGCG ATG GCG GAG GAA TTT GGA AGC ATA G-3’ and 5’-GGGG ACAACTTTGTATAATAAAGTTGG GTG ATA TTA CCT GAA GTT ATG CCT CAA AAT C-3’ and subcloned in pDONR P2R-P3 vector. The GFP sequence was amplified from pSITE-2CA vector using primers 5`-CACCATGGTGAGCAAGGGCGAGGAGCTGT-3`and 5`-CTTGTACAGCTCGTCCATGCCGAGAG-3`then cloned into pENTR/D-TOPO (Invitrogen, Life Technologies). The three fragments were recombined in pK7m34GW vector [27] using Multisite Gateway technology (Invitrogen, Life Technologies). This construct was transferred to *Agrobacterium tumefaciens* GV3101::pMP90 by the freeze-thaw method [28]. This construct was transferred to Arabidopsis *bzip60* (SAIL_283_B03) insertional mutant plants by floral dip method [29]. Plants harbouring the transgen were selected on 0.5x MS plates containing kanamycin.

### Chemical, abiotic and hormone stresses

For the time-course analyses of the splicing of bZIP60, 7-days-old Arabidopsis seedlings were grown in liquid Murashige and Skoog (MS) medium (0.5x MS salts, 1% sucrose). To evaluate heat stress, the flasks containing plants were submerged in a water bath at 42°C for the indicated time. To test salt and osmotic stress, seedlings were treated with liquid MS medium containing 150 mM NaCl (salt stress) or 300 mM Mannitol (osmotic stress) for the indicated times. To evaluate the role of hormones associated to stress, seedlings were treated with liquid MS medium, containing SA (0.5 mM) or ABA (10 μM) for the indicated times. For treatments with chemicals, seedlings were incubated with liquid MS medium containing Tm (5 μg/mL) or DTT (2 mM) for the indicated times. The same concentrations were used for the washing-out experiments and room temperature (RT) in the heat stress wash-out experiment. To evaluate the splicing level in roots, 7-day-old Arabidopsis seedlings were grown in MS solid medium (0.5x MS salts, 1% sucrose, 0,4% phytagel) in a vertical position. Then plants were transferred to MS solid medium plates supplemented with Tm (5 μg/mL) or DTT (2 mM) for the indicated time. The splicing level in different organs, with and without heat treatment, was evaluated on six-week-old plants grown in hydroponic media exposed to 42°C for 1 hour.

### RT-PCR and bZIP60 splicing assay

Arabidopsis seedlings or different organs were harvested in liquid nitrogen. RNA was extracted from seedlings and organs using TRIzol reagent (Invitrogen, Life Technologies) or Plant RNA Reagent (Invitrogen, Life Technologies) respectively. Subsequently total RNA was quantified using a Qubit fluorometer (Invitrogen, Life Technologies) and analyzed by CE-LIF (AATI Fragment Analyzer, Advanced Analytical Technologies) using STD SENSITIVITY TOTAL RNA ANALYSIS KIT (Advanced Analytical Technologies) to evaluate the RQN (RNA Quality Number). Total RNA was treated with DNase I (Fermentas, Thermo Scientific) and cDNA was synthesized using a SuperScript II first-strand RT-PCR kit (Invitrogen, Life Technologies). To amplify both the unspliced and spliced forms of bZIP60, the following primers were used: 5`-GCTGAAAACCAGTCTCTACGTT-3`and 5`-AAGCAGGGAACCCAACAG-3`. PCR conditions for amplification were: initial denaturation: 5 min at 95°C; 30 s at 95°C, 30 s at 58°C and 30 s at 72°C during 34 cycles. Subsequently, PCR products were resolved by gel electrophoresis on agarose (3.5% w/v) using TAE 1X as running buffer or prepared for CE-LIF using the High Sensitivity NGS Fragment Analysis Kit (Advanced Analytical Technologies) and analyzed in the fragment analyzer instrument (AATI Fragment Analyzer, Advanced Analytical Technologies) following the manufacturer instructions.

### *in vitro* transcription

We proceeded to amplify by PCR the processed form of bZIP60 cloned into pGEM-T vector (Promega) using the 5`-CAG AGA TGC ATA ATA CGA CTC ACT ATA GGG AGA CCA CCA TGG CGG AGG AAT TTG GAA GC-3`and 5`-T(x30) TCA CGC AAG GGT TAA GAT-3`primers. The PCR product was resolved by gel electrophoresis on agarose and purified using the GeneJET gel extraction Kit (Fermentas, Thermo Scientific). Then the purified PCR product was used for *in vitro* transcription using the T7 RiboMAX Large Scale RNA Production Systems (Promega) following the manufacturer instructions, except for the incubation time which was extended to 4 hours. RNA from the *in vitro* transcription was purified using RNeasy Plus Micro kit (QIAGEN). Purified RNA was quantified using the Qubit RNA BR Assay Kit (Life Technologies) and Qubit fluorometer (Life Technologies). Then, we transformed the amount of RNA to number of molecules using the following formula:

$$number\;of\;copies\;\left(RNA\;molecules\right)=\frac{X\;ng*6.0221*10^{23}\;molecules/mole}{\left(N*330\;g/mole\right)*1*10^{9}ng/g}$$ 1

where *X* is the amount of RNA in nanograms and *N* represent the length of a RNA molecule in bases.

### bZIP60 splicing quantification

In order to determine the range of detection of the CE-LIF, cDNA was synthesized using different quantities of bZIP60s RNA generated *in vitro* and subsequently amplified as described previously. PCR products were resolved by CE-LIF following the same procedure described in the previous section. From this analysis, we determined the linear range of detection for the CE-LIF and a standard curve was constructed using the peak area data obtained from the amplification of 1 to 1000 copies of bZIP60s. To determine the number of copies of bZIP60s on the biological samples, the value of the peak area, corresponding to the processed form of bZIP60, was interpolated into the standard curve. Then we applied the following formula:

$$bZIP60S\;\left(N{}^{\circ}\;copies\;per\;ug\;total\;RNA\right)=\left(\frac{N{}^{\circ}\;bZIP60s\;copies*\;DF\;cDNA}{total\;RNA\;input\;\left(in\;micrograms\right)}\right)-bkg$$ 2

where *DF cDNA* correspond to the dilution factor of the volume of cDNA used and *bkg* is the number of bZIP60s copies determined using genomic DNA (gDNA) as template.

### Confocal microscopy

Detection of the GFP-tagged bZIP60 protein was performed using a FV1000 laser scanning confocal microscope (Olympus, USA). Seven days-old seedlings, treated or not treated with chemicals that trigger UPR were stained with FM 4–64 dye (Molecular Probes, Life Technologies) diluted on liquid MS media during 5 minutes at room temperature. After staining, seedlings were rinsed on liquid MS media, mounted on water and roots observed under the confocal microscope using a 20X objective. Fluorescence emission was obtained by laser excitation of GFP and FM 4–64 at 488 nm. Emission was collected in the range 500–600 and 655–755 nm, respectively. In order to visualize the nucleus of roots from transgenic plants, a similar procedure as described above was performed but instead of staining seedlings with FM 4–64, we used the 4′, 6-diamidino-2-phenylindole (DAPI) dye (Molecular Probes, Life Technologies) diluted in 1x PBS buffer with 1% v/v Triton X-100 for 10 min at room temperature. After staining, seedling were washed with 1x PBS, mounted on water and observed under the confocal microscope using a 20X objective. Fluorescence emission was obtained using laser excitation of 405 nm for DAPI and 488 nm for GFP using sequential scanning mode to eliminate crosstalk and signal bleed-through. The emission signal was collected in the range 425–475 and 500–600 nm, respectively. For subcellular imaging, seedlings of 7 days-old treated with DMSO, DMSO plus DTT, MG132 or MG132 plus DTT were stained with ER-Tracker Blue-White DPX dye (Molecular Probes, Life Technologies) diluted on liquid MS media during 45 minutes, rinsed on liquid MS media and stained with FM 4–64 dye (Molecular Probes, Life Technologies) during 5 minutes; at room temperature. After staining, seedlings were rinsed on liquid MS media, mounted on water and roots observed under the confocal microscope using a 60X objective. The emission signals for ER-Tracker, GFP and FM 4–64 were acquired using sequential scanning mode to eliminate crosstalk and emission signal bleed-through. Fluorescence emission was obtained using laser excitation of 405 nm for ER-Tracker and 488 nm for GFP/FM 4–64, the emission was collected in the range 425–475, 500–600 and 655–755 nm, respectively.

### Immunoblot analysis

For immunoblot analysis, seedlings or roots of 7 days-old plants were homogenized in SDS loading buffer (250 mm Tris-HCl, 10% SDS, 25% glycerol, 0.1% bromophenol blue, 10%β-mercaptoethanol; [30]) using 50 μL of buffer for 10 mg of tissue. Then samples were heated at 75°C for 15 min and supernatants collected by centrifugation. A 10 μL aliquot was analyzed by SDS-PAGE [31] and immunoblotting [32]. Immunoblots were probed with a 1:5000 dilution of the anti-GFP antibody (Abcam Ab290), followed by incubation in a 1:10000 dilution of a horseradish peroxidase-conjugated goat anti rabbit antibody (KPL 074–1506). Equal loading was confirmed by stripping and re-probing the immunoblots in a 1:10000 dilution of anti-PEPC (Abcam ab34793). The secondary antibody was visualized by SuperSignal West Pico Chemiluminescent Kit (Thermo Scientific, Rockford, IL, USA) and exposed on CL-XPosure Film (Thermo Scientific, Rockford, IL, USA).

## Results

### The use of CE-LIF allows a higher sensitivity in the assessment of bZIP60 splicing

**Fig 1:**
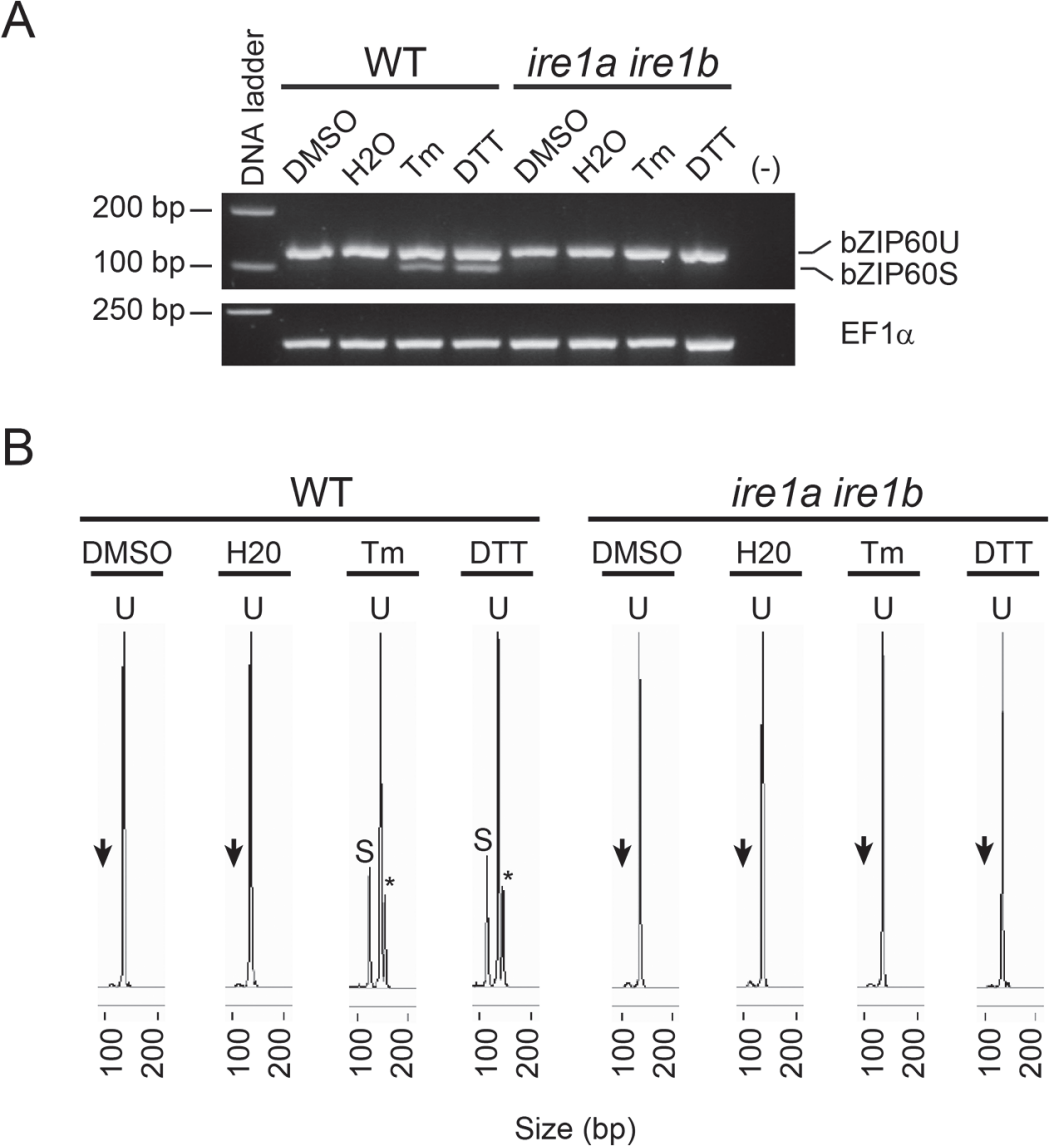
Detection of bZIP60 mRNA variants using capillary electrophoresis coupled to laser induced fluorescence (CE-LIF). (A) Agarose gel electrophoresis (3.5% p/v) of RT-PCR products obtained from total RNA extracted from wild type (WT) and *ire1a ire1b* mutant Arabidopsis seedlings (7-days-old) treated with DTT (2 mM) or tunicamycin (Tm; 5 μg/mL). DMSO (0.2% v/v) and water (H2O) treated samples served as mock control for the chemicals. Elongation factor 1 alpha (EF1α) expression served as a control. A PCR reaction without cDNA was performed as negative control. Data are representative of three independent experiments. (B) CE-LIF analysis of the same RT-PCR products mentioned above. The peaks from the spherograms corresponding to the unspliced form of bZIP60 (U) and spliced form (S) are shown. Black arrows indicated the presence of a small peak in mock samples (DMSO and water) from both genotypes as well in DTT and Tm samples from *ire1a ire1b* mutant. Asterisks indicate the detection of a third peak, only present in DTT and Tm samples obtained from wild type plants. Data are representative of three independent experiments.

The splicing of bZIP60 was evaluated by amplifying the double hairpin region of bZIP60 using PCR and total RNA obtained from plants, followed by separation of the amplicons using agarose gel electrophoresis. As expected, treatment of wild type plants with DTT and tunicamycin (Tm) produced two amplicons. The one that exhibited the higher electrophoretic mobility corresponded to the spliced form of bZIP60, as it has been already reported [15,16,17]. In order to set a method that may determine with higher sensitivity the splicing of bZIP60, we decided to use capillary electrophoresis with laser-induced fluorescence (CE-LIF), a procedure that also allows to detect both the spliced and unspliced forms in the same assay. Therefore, samples already analyzed by agarose gels (Fig 1A) were re-assessed using CE-LIF. These analyses showed a single peak in the control, corresponding to the unspliced form of bZIP60 (U) (Fig 1B). When plants were treated with DTT and Tm, a second peak was also observed. This second peak was not present on *ire1a ire1b* mutant plants, indicating that this peak corresponded to the spliced form of bZIP60 (bZIP60s). On samples where the splicing of bZIP60 was induced by DTT and Tm, a third peak was observed under these conditions. This is a fragment with a molecular weight higher than the spliced and unspliced forms and likely corresponds to the hybrid molecule formed by the annealing of the spliced and unspliced forms of bZIP60, as it has been described previously [17]. The high sensitivity of CE-LIF allowed us to observe a peak, almost undetectable on *ire1a ire1b* mutant plants, migrating at the same retention time that the bZIP60 spliced form. Intriguingly, this was present in all conditions, including the controls. We hypothesized that this is an artifact, likely due to the complex secondary structure of the double hairpin present in bZIP60. In order to confirm this assumption, we performed PCR amplification of genomic DNA as template, a sample that should contain no spliced molecules. The analysis of the genomic DNA also produced the same signal (S1 Fig). Therefore, this signal was considered background and was subtracted from all the analyses presented in this work as described in materials and methods section.

**Fig 2:**
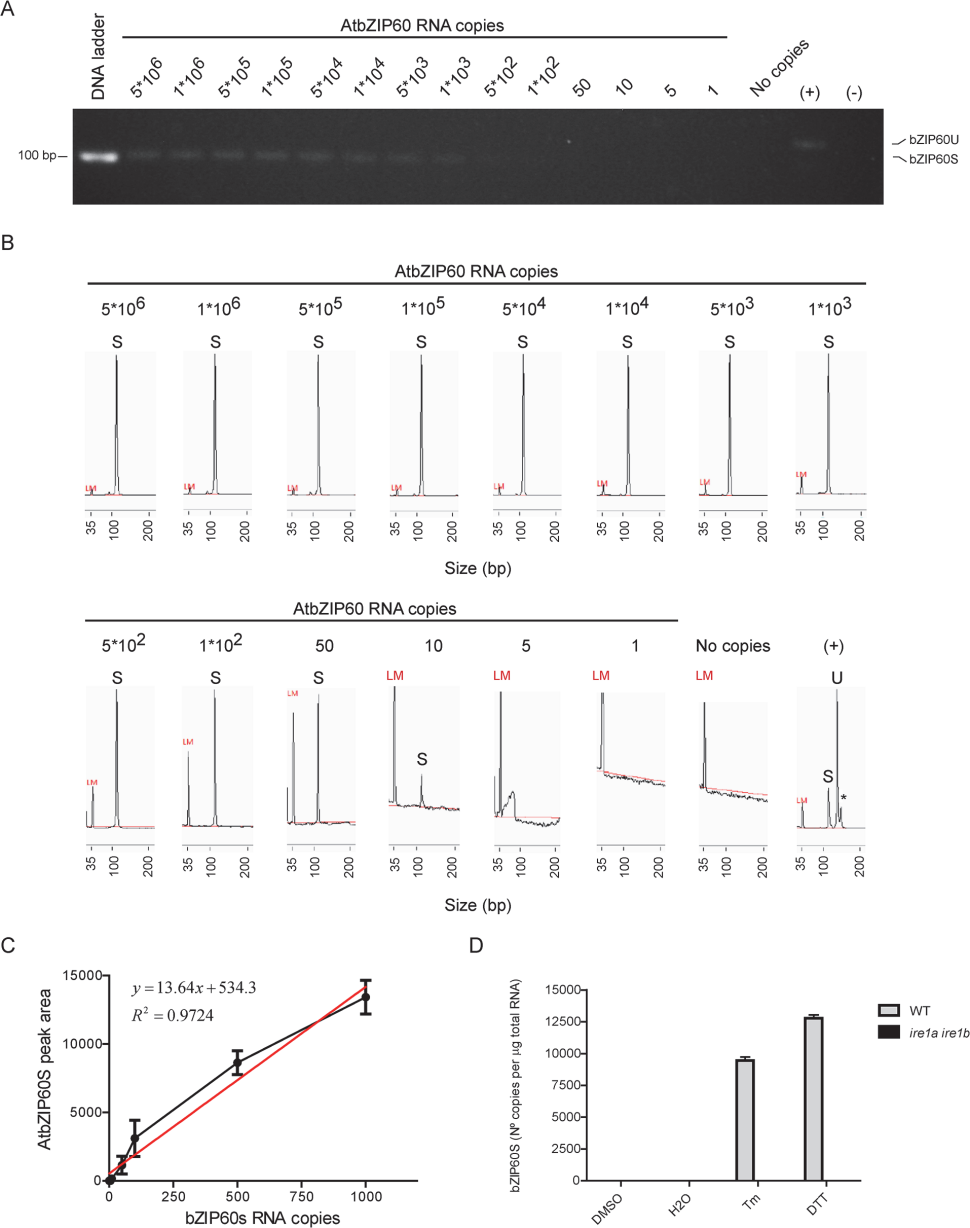
Setting parameters for quantification of bZIP60S number of copies using CE-LIF. In order to determine the range of detection of CE-LIF in comparison to agarose gel electrophoresis, we performed RT-PCR using serial dilutions of bZIP60s RNA generated *in vitro* as template. Then RT-PCR products were resolved by agarose gel electrophoresis (3.5% p/v) (A) or CE-LIF (B). A PCR reaction with cDNA from Arabidopsis seedlings (7-days-old) treated with DTT (2 mM) was performed as positive control. A PCR reaction without cDNA was performed as negative control. LM, refers to Low Marker DNA fragment (35 base pairs) used by the CE-LIF instrument as internal standard. Data are representative of three independent experiments. (C) Graph for the number of copies of bZIP60s (1 to 1000 copies) vs the peak area data for bZIP60s. The linear range of detection was determined from the above data and used to make the standard curve. Data are representative of three independent experiments. (D) Quantification of the number of copies of bZIP60s using CE-LIF from samples of wild type (WT) and *ire1a ire1b* mutant Arabidopsis seedlings (7-days-old) treated with DTT or tunicamycin. DMSO (0.2% v/v) and water (H2O) treated samples served as mock control for the chemicals. All quantification calculations were performed as described in the materials and methods section. Data are representative of three independent experiments. Data are shown as mean ± SD.

In order to quantify the amount of molecules that can be detected by agarose gels and the CE-LIF method, we synthesized *in vitro* the spliced form of bZIP60 and serial dilutions were made in order to determine the minimal number of molecules that can be detected by both methods. The agarose gel method showed that the lowest number that can be detected is between 500–1,000 molecules (Fig 2A), whereas the CE-LIF method was able to detect an amount as low as 10 molecules of the spliced form of bZIP60 upon the amplification by PCR (Fig 2B). Therefore, CE-LIF showed 50 to 100-fold higher sensitivity than the agarose gel method. Once we set the quantitative procedure based on CE-LIF (Fig 2C), we determined that treating plants for two hours with DTT and Tm produced 9,460 copies/μg of total RNA and 12,768 copies/μg of total RNA respectively (Fig 2D).

### bZIP60 processing is attenuated under continuos exposure to ER stress

**Fig 3:**
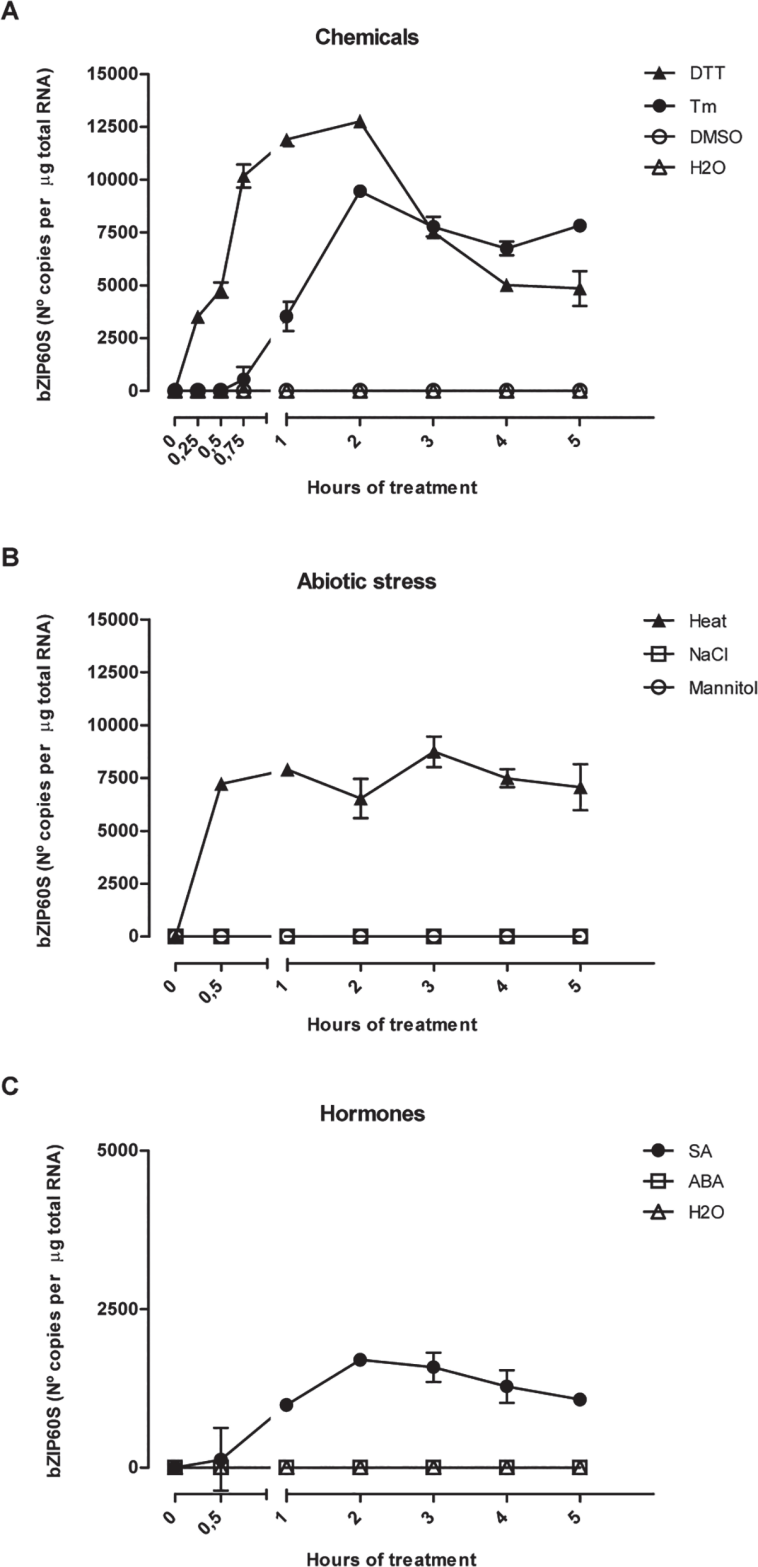
The magnitude of bZIP60 processing depends on stimulus and time. (A) CE-LIF analysis for products of RT-PCR obtained from total RNA extracted from wild type Arabidopsis seedlings (7-days-old) treated with DTT (2 mM) or tunicamycin (Tm; 5 μg/mL) at the periods of time indicated. DMSO (0.2% v/v) and water (H2O) treated samples served as mock control for the chemicals. (B) CE-LIF analysis for RT-PCR products obtained from total RNA extracted from wild type Arabidopsis seedlings (7-days-old) treated with NaCl (150 mM), mannitol (300 mM) or exposed to high temperature (Heat; 42°C) at the periods of time indicated. (C) CE-LIF analysis for products of RT-PCR obtained from total RNA extracted from wild type Arabidopsis seedlings (7-days-old) treated with SA (0.5 mM) or ABA (10 μM) at the periods of time indicated. Water (H2O) treated samples served as mock control for the chemicals. All quantification calculations were performed as described in materials and methods section. Data are representative of three independent experiments. Data are shown as mean ± SD.

Then we analyzed the time-course of the splicing of bZIP60, after treatment with these two inducers of UPR (Fig 3A). Upon using DTT, we observed that after 15 minutes of treatment, we detected the spliced form of bZIP60, which increased during the time-course of the treatment, reaching a maximum at 2 hours. After that time, the amount of the spliced form was reduced, decreasing its content to near half of the maximum value. When plants were incubated with Tm, the spliced form of bZIP60 was detected after 45 min of incubation, reaching a maximum at 2 hours. Then the content decreased, but to a lesser extent in comparison to samples treated with DTT. In order to check whether the chemicals became inactive during the time-course experiment, we took culture media from plants incubated with DTT or Tm and we added it to a batch of untreated plants. The results showed that culture media taken from plants treated for at least 2 hours with these compounds, were still able to induce the splicing of bZIP60, indicating that DTT and Tm were still active (S2 Fig). Therefore, the decrease of the amount of the spliced form observed after 2 hrs of treatment with DTT or Tm is not due to the inability of these compounds to induce the splicing of bZIP60.

It has been shown that splicing of bZIP60 is also triggered by heat [15,17]. Therefore, we performed a time-course experiment, exposing the plants to heat (Fig 3B). Analyses at 30 min showed that splicing of bZIP60 produced around 7,500 copies/μg of total RNA. This number did not changed significatively and it was kept throughout the 5 hours of treatment. Other abiotic stresses, such as salt (NaCl) or osmotic (mannitol), produced no detectable splicing of bZIP60. Furthermore, it has been reported that treatment with salicylic acid also leads to splicing of bZIP60 [17,18]; therefore, we performed a time-course analysis of the effect of salicylic acid on the splicing of bZIP60. The spliced form of bZIP60 was first observed at 30 min reaching a maximum of 1,700 copies/μg of total RNA after 2 hrs of treatment (Fig 3C). After that time, it decreased to 1,000 copies/μg of total RNA at 5 hours of treatment. To test if ABA, a stress-related hormone, is involved in triggering the UPR under abiotic stress conditions, we determined the splicing of bZIP60 upon treatment with ABA. No effect of ABA on the splicing of bZIP60 was observed (Fig 3C).

### The response of Arabidopsis plants to the re-induction of UPR depends on the stimulus

**Fig 4:**
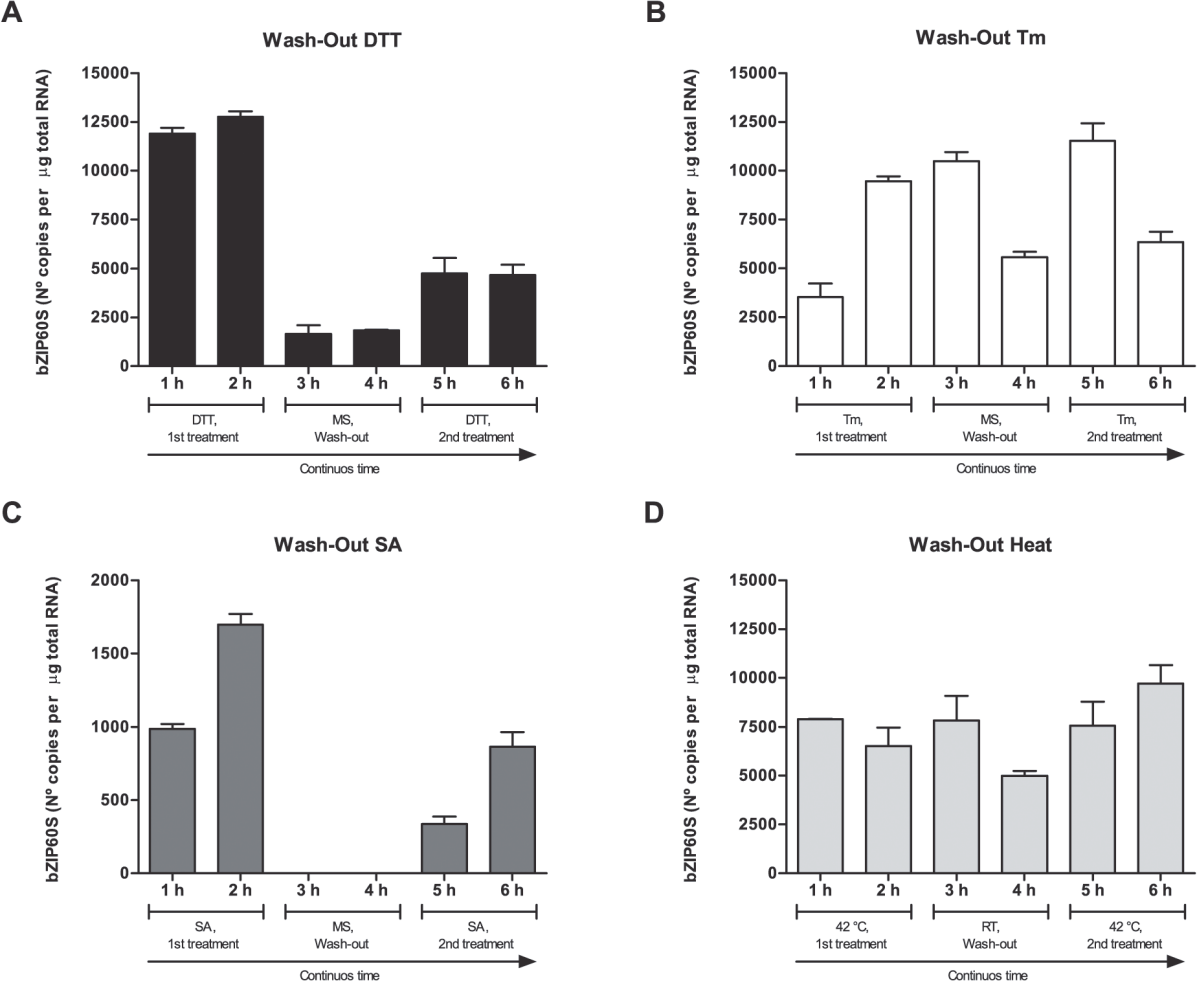
The processing of AtbZIP60s is a dynamic process. (A) Wild-type Arabidopsis seedlings (7-days-old) were treated with DTT (2 mM) for two hours then media was changed to fresh liquid 0.5x MS plus 1% sucrose for two additional hours and finally treated with DTT (2 mM) for another two hours. The complete experiment was extended for six hours. Total RNA samples were obtained at the indicated time points. RT-PCR products generated from total RNA were analyzed by CE-LIF. Identical procedures were performed for treatments with tunicamycin (Tm; 5 μg/mL) (B) or salicylic acid (SA; 0.5 mM) (C). In the case of heat treatment, instead of changing the culture media, flasks with seedlings exposed to high temperature (42°C) were transfered to room temperature (RT) for two hours and re-exposed to high temperature (42°C) during two more hours (D). All calculations were performed as described in materials and methods section. Data are representative of three independent experiments. Data are shown as mean ± SD.

We analyzed the return to baseline of the content of the spliced form of bZIP60 by washing-out the effector and determining the content of the spliced form of bZIP60. Replacement of DTT with fresh media containing no DTT led to a significant decrease in the amount of the spliced form of bZIP60 after one hour. After two hours of washing out DTT, we added it back and analyzed the splicing after one and two hours. We observed an increase in the splicing but the maximum reached in this second treatment was less than half of the initial content (Fig 4A). The wash-out of Tm produced a slower decrease in the content of the spliced form of bZIP60 compared to DTT. Moreover, adding back Tm brought the spliced form of bZIP60 to a level similar to that observed after one hour of the initial treatment; however, there was a decrease after two hours of re-treatment (Fig 4B). The wash-out of SA led to a complete loss of the spliced form of bZIP60. The re-addition of SA led to a partial recovery of the levels of the spliced form of bZIP60 detected after the initial treatment with SA (Fig 4C). Finally, reducing the temperature from 42°C to 22°C produced a slight decrease in the amount of the spliced form after two hrs. The return to 42°C led to a recovery in the amount of the spliced form, which after 2 hrs was even higher (Fig 4D). Overall, these results show that the processing of bZIP60 is a highly dynamic process that depends on the the stimulus applied to the plant.

### The lack of bZIP17 induces a higher splicing of bZIP60 upon treatment with salicylic acid

The splicing of bZIP60 mediated by IRE1 is only one of the branches of the UPR signaling pathways in plants. There are two other pathways that are also activated in response to ER stress. These are mediated by the transcription factors bZIP28 and bZIP17. In order to evaluate the extent to which the splicing of bZIP60 is affected by the presence or absence of these transcription factors, we evaluated the level of splicing of bZIP60 on mutants on bZIP17 and bZIP28 when plants were exposed to different stimuli that trigger the splicing of bZIP60. The results showed that mutants in bZIP17 exposed to SA exhibited an increment in the level of splicing of bZIP60 (S3 Fig). All the other treatments produced no differences between wild type and mutant plants.

### The level of splicing of bZIP60 changes in different organs

**Fig 5:**
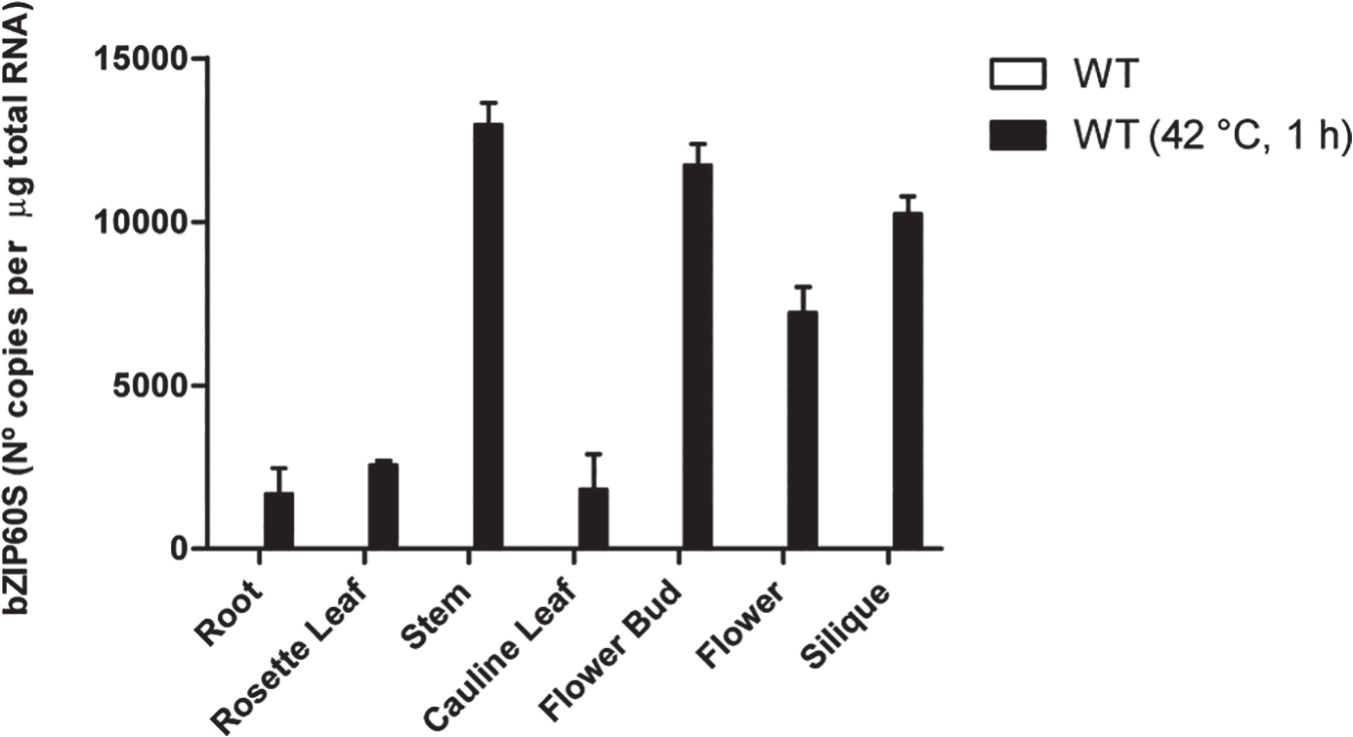
The processing of bZIP60 is a tissue-specific process. Six weeks-old Arabidopsis plants of were exposed or not, to high temperature (42°C) during 1 hour. Then total RNA was extracted from the indicated tissues. RT-PCR products generated from total RNA isolated were analyzed by CE-LIF. All calculations were performed as described in the materials and methods section. Data are representative of three independent experiments. Data are shown as mean ± SD.

In order to evaluate whether the splicing of bZIP60 occurs in all organs when the plant is exposed to ER stress, we analyzed the splicing of bZIP60 in different organs when the plant was exposed to heat, a strong inducer of bZIP60 processing. The results showed that splicing occurred in all organs analyzed (Fig 5); however, the extent of the splicing varied being highest in flower buds, stems and siliques and the lowest in rosette leaves, root and cauline leaves. Under these conditions we were not able to detect endogenous splicing in any of these organs.

### GFP-AtbZIP60 accumulate in the nucleus upon treatment with DTT and Tunicamycin

**Fig 6:**
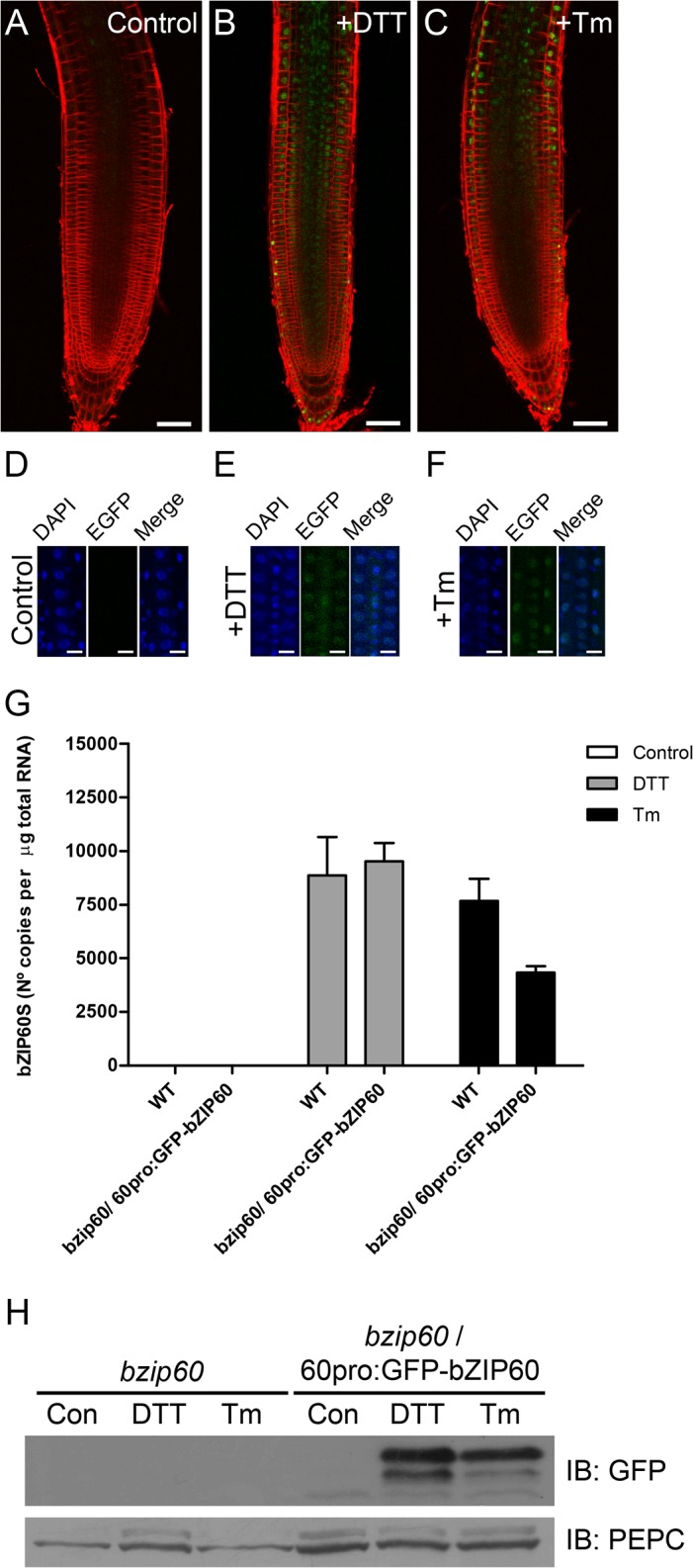
GFP-bZIP60s protein accumulation in the nucleus correlates with the processing of bZIP60 under ER stress. (A-C) Seedlings of transgenic Arabidopsis plants harbouring the construct 60pro:GFP-bZIP60 were treated with DTT (2 mM) or tunicamycin (5 μg/mL) during 3 hours. After the treatment, seedlings were stained with FM 4–64 (5 μM) during 5 minutes at room temperature and the roots analyzed by confocal microscopy. Untreated seedlings were used as control. Scale bar equals 50 μm. Images are representative of three independent experiments. (D-F) Seedlings of transgenic Arabidopsis plants harbouring the construct 60pro:GFP-bZIP60 were treated as mentioned above. After the treatment, seedlings were stained with DAPI (1 μg/mL) during 15 minutes at room temperature and the roots analyzed by confocal microscopy. Untreated seedlings were used as control. Scale bar equals 15 μm. Images are representative of three independent experiments. (G) CE-LIF analysis of the products of RT-PCR obtained from total RNA extracted from roots obtained from wild-type (WT) plants or *bzip60*/ 60pro:GFP-bZIP60 transgenic Arabidopsis (7-days-old), treated with DTT (2 mM) or tunicamycin (Tm; 5 μg/mL) during 3 hours. Untreated seedlings were used as control. All calculations were performed as described in the materials and methods section. Data are representative of three independent experiments. Data are shown as mean ± SD. (H) Immunoblot analysis of total protein extracts obtained from roots derived from *bzip60*/ 60pro:GFP-bZIP60 transgenic Arabidopsis plants (7-days-old) treated with DTT (2 mM) or tunicamycin (Tm; 5 μg/mL) during 3 hours, using a polyclonal antibody against GFP. *bzip60* mutant roots were used as control. Anti-PEPC antibody was used as loading control. Data are representative of three independent experiments.

Throughout all the experiments shown in this work, we have assessed the splicing of bZIP60 at the transcriptional level. However, we do not know whether the splicing of bZIP60 necessarily correlates with the appearance of the protein in the nucleus. Therefore, in order to detect the subcellular distribution of the protein at single cell levels, we decided to use a GFP-tagged version of bZIP60. This approach provides enhanced resolution in comparison to using antibodies against the endogenous protein, a procedure that in addition, in plant tissues is rather cumbersome. Furthermore, in order to ensure the proper expression of the protein, we generated a GFP-tagged version of bZIP60 under the control of its own promoter, a procedure that should lead to protein levels similar to the wild type condition. This construct was utilized to produce transgenic plants in a *bzip60* mutant background and several independent lines were selected. Untreated plants showed no fluorescence, suggesting that the content of GFP-AtbZIP60 is very low and not detectable in our conditions (Fig 6A). Upon treatment with DTT or tunicamycin, a strong signal was observed in the nucleus (Fig 6B and 6C). In order to confirm that the GFP signal came from the nuclei, we checked whether upon treatments that trigger the UPR, the GFP signal co-localized with the signal generated by DAPI (4', 6-diamidino-2-phenylindole), a fluorescent dye that binds DNA. In accordance with our previous observations we did not detect the GFP signal in untreated transgenic plants (Fig 6D). Also, we confirmed that the GFP and DAPI signals colocalize under DTT and tunicamycin treatments (Fig 6E and 6F). To correlate the GFP signal in the nucleus with the splicing of bZIP60, we analyzed the accumulation of the spliced form of bZIP60 in the transgenic plants by CE-LIF. The results showed that splicing of the bZIP60 mRNA occurred to a similar extent both in the wild type and the transgenic line (Fig 6G). This observation indicates that splicing of the bZIP60 mRNA correlates with the accumulation of the bZIP60 protein in the nucleus. Intriguingly, the plants expressing GFP-bZIP60 showed no fluorescence under basal conditions. The lack of fluorescence in the absence of any treatment suggests that GFP signal in these conditions is below the detection level, the fusion protein is absent or the protein suffered a change in its conformation leading to no fluorescence. To test whether any of these possibilities is true, we performed immunoblot analyses of proteins extracted from roots treated with and without chemicals that induce UPR. The results showed that a protein with an electrophoretic mobility that accounts for the molecular mass of bZIP60 plus GFP was detected only when plants were treated with DTT or Tm (Fig 6H). No protein was detected in the absence of the UPR inducers suggesting that the protein derived from the unspliced mRNA of bZIP60 (bZIP60u) was absent or undetectable.

### GFP-bZIP60u protein levels appear to be controlled by the proteasome

**Fig 7:**
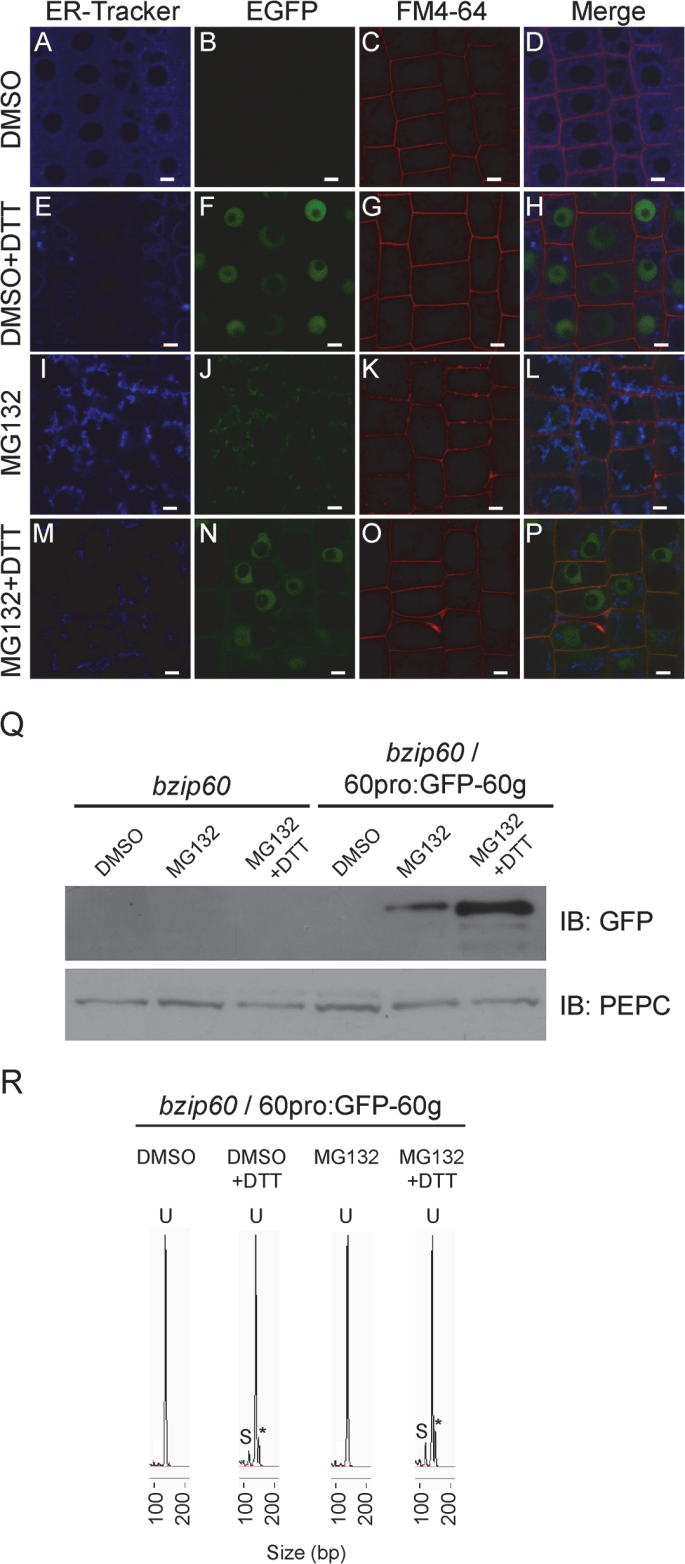
Inhibition of the proteasome activity leads to the detection of GFP-bZIP60u on the ER. Seedlings (7-days-old) of transgenic Arabidopsis plants harbouring the construct 60pro:GFP-bZIP60 were pretreated with DMSO (0.2% v/v) or MG132 (100 mM) during 18 hours. Then seedlings were treated with DMSO (0.2% v/v) (A-D), DMSO (0.2% v/v) plus DTT (2 mM) (E-H), MG132 (100 μM) (I-L) or MG132 (100 μM) plus DTT (2 mM) (M-P) during 3 hours. All treatments were performed in 0.5x MS phytagel plates supplemented with the chemicals mentioned above at the indicated concentrations. After treatment, seedlings were stained with ER-Tracker Blue-White DPX (1 μM) during 45 minutes, rinsed and stained with FM 4–64 (5 μM) during additional 5 minutes at room temperature. Finally, roots were analyzed by confocal microscopy. Scale bar equals 5 μm. Images are representative of three independent experiments. (Q) Immunoblot analysis of total protein extracts obtained from *bzip60*/ 60pro:GFP-bZIP60 transgenic Arabidopsis seedlings (7-days-old) treated with DMSO (0.2% v/v), MG132 (100 μM) or MG132 (100 μM) plus DTT (2 mM) as mentioned above, using a polyclonal antibody against GFP. *bzip60* mutant seedlings were used as control. Anti-PEPC antibody was used as loading control. Data are representative of three independent experiments. (R) CE-LIF analysis of RT-PCR products obtained from total RNA extracted from roots of *bzip60* mutant or *bzip60*/ 60pro:GFP-bZIP60 transgenic Arabidopsis plants (7-days-old) treated with DMSO (0.2% v/v), DMSO (0.2% v/v) plus DTT (2 mM), MG132 (100 μM) or MG132 (100 μM) plus DTT (2 mM) as described above. Spherograms peaks, corresponding to the unspliced form of bZIP60 (U) and spliced form (S), are depicted. Asterisks indicate the detection of a third peak only present in DTT treated samples. Data are representative of three independent experiments.

The previous results suggest that bZIP60u is regulated either at the transcriptional or posttranslational level under basal conditions. However, since we detected the mRNA corresponding to the unspliced form (S4 Fig), we thought that regulation might be occurring by proteolysis. It is well known that the proteasome is involved in a number of regulatory processes by degrading proteins; therefore, we decided to check whether this complex is playing a role in the degradation of bZIP60u by using MG132, a proteosome inhibitor, thus, the transgenic line expressing GFP-bZIP60 was treated with MG132 and the fluorescence was analyzed by confocal microscopy. The results showed again that GFP-bZIP60 was absent under basal conditions (Fig 7B); however, after using MG132 we were able to detect fluorescence (Fig 7J). The pattern was different from the pattern observed when plants were treated only with DTT, where a nuclear localization was observed (Fig 7F). Since it has been claimed that bZIP60u is located in the ER [23], we used an ER-tracker to find out whether the fluorescence was located in the endoplasmic reticulum. Fig 7J shows that an important portion of the GFP signal seemed to have a localization similar to that of the ER-tracker (Fig 7I). In order to evaluate the effect of DTT on plants treated with MG132 we incubated plants with both chemicals and we analyzed the plants using confocal microscopy. The fluorescence pattern changed in comparison to the images obtained from plants treated only with MG132, since the signal was now more prominent in the nucleus (Fig 7N). However, some fluorescence signal was still observed in the cytoplasm.

The results described above suggested that GFP-bZIP60u protein was present when plants were treated with MG132. To check this out, immunoblot analyses were performed and they indicated the presence of a protein in extracts from plants treated with MG132 (Fig 7Q). In contrast, no protein was detected on samples not treated with MG132. To rule out that the protein detected was produced by the spliced form of bZIP60 mRNA, we analyzed whether MG132 induces the splicing of this mRNA by using CE-LIF. The results indicated that no splicing occurred under this condition (Fig 7R), suggesting that the protein observed corresponds to bZIP60u. Finally, the addition of MG132 plus DTT resulted in the presence of a more abundant protein, with a MW similar to the protein observed only on the presence of MG132, which may correspond to the spliced form of the protein.

## Discussion

The splicing of bZIP60 has been assessed by amplifying the spliced and unspliced forms of bZIP60, followed by the separation of the amplicons by agarose gel electrophoresis [15,16,17]. This has been a useful method; however, it is not sensitive enough to assess quantitatively the changes in the content of bZIP60s. Then, we set up a new method using CE-LIF to assess the splicing of bZIP60. This method allowed us to get higher sensitivity under conditions where we can detect both the spliced and the unspliced forms of bZIP60. This is an advantage in comparison to Q-PCR, another high-sensitive method, used to assess the splicing of bZIP60. Our analyses showed that the unspliced form was always present at saturating levels and it was not possible to set a condition where both the spliced and unspliced forms were present at not saturating conditions. In addition, our results indicate that under all conditions where the splicing of bZIP60 was assessed, the unspliced form was always present at higher levels in comparison to the spliced form, suggesting that in plants, only a minor fraction of the bZIP60 mRNA undergoes splicing. This result may be explained by a lower activity of IRE1 in plants or by the existance of different pools of the bZIP60 mRNA that can be accessed by IRE1.

The assessment of different stimuli that trigger UPR showed that DTT, Tm, heat and SA were able to induce the splicing of bZIP60. In contrast, NaCl, mannitol, ABA, water and DMSO had no effect on the induction of splicing of bZIP60. Among the inducers, DTT had the greatest effect, followed by Tm, heat and SA. Interestingly, both DTT and SA alter the redox balance of the cell and the dynamic of the splicing and its recovery is similar, upon the addition of both compounds. On the other hand, the dynamics observed upon treatment with Tm and heat shows some intriguing similarities. A hypothesis to explain this phenomenon lies on the fact that both produce severe effects on protein folding, since Tm eliminates the N-glycan present in glycoproteins, affecting the folding assisted by the ER quality control. On the same line, heat can induce the formation of protein aggregates by stimulating the interaction of hydrophobic domains present in the protein, leading to abnormal folding states that can not be recovered.

Since bZIP60 is one of the three transcription factors that are related to the unfolded protein response in plants, we wonder whether the lack of any of the others (bZIP17 and bZIP28) could increase the splicing of bZIP60. Surprisingly, no compensatory mechanisms were activated with the exception of the bZIP17 mutant treated with SA. This suggests that the effect of SA on the splicing of bZIP60 is somehow linked to the function of bZIP17.

Our data indicate that splicing of bZIP60 does not occur to the same extent in different organs. This suggests that the signalling pathway, triggered by the spliced form of bZIP60, is more important on certain plant organs. Our method allowed us to determine levels as low as 100 copies/μg of total RNA. Under these conditions we were unable to detect splicing of bZIP60 on untreated plants. However, we cannot rule out the possibility that splicing of bZIP60 may occur endogenously at even lower levels on the organs analyzed. One of the UPR components that play an essential role in the splicing of bZIP60 is IRE1. No tissue specific analysis of the transcript levels of IRE1A and IRE1B have been reported yet in plants. However, the analyses of public data bases such as GENEVESTIGATOR [33] and eFP Browser [34] show that both IRE1A and IRE1B are differentially expressed in different organs, suggesting that splicing of bZIP60 can occur at different levels in different organs depending on the expression of IRE1. In any case, the correlation between the IRE1 levels and the splicing of bZIP60 needs further experimental evaluation.

Even though we were able to measure the levels of the spliced form of bZIP60 under ER stress induced by different stimuli, we cannot assume that a correlation between an increasing amount of the spliced form of the bZIP60 mRNA and the subsequent accumulation of the protein derived from this transcript, may occur in the nucleus. In order to address this issue we generated a transgenic line, expressing a GFP-tagged version of bZIP60 under the control of the endogenous promoter, in the *bzip60* mutant background. These transgenic plants showed that treatment with DTT and Tm produced an accumulation of this protein in the nucleus, confirming that treatments leading to high levels of the spliced form also lead to an accumulation of the protein in the nucleus. Interestingly, our transgenic plants showed no detectable GFP fluorescence in the absence of any treatment. In addition, no protein was observed by immunoblot analyses, suggesting that no bZIP60 protein is present at basal conditions. However, the transcript encoding for the unspliced form was present under this condition; thus, this results poses a question about the stability of the bZIP60 protein under basal conditions. Regarding the stability of the unspliced form in the plants, Deng et al. [15], showed that expression of a tagged version of the unspliced form of bZIP60 could be detected in tobacco BY-2 cells. This experiment was carried out by expressing the gene encoding the GFP-bZIP60 fusion under the control of the 35S promoter. In contrast, we expressed the GFP-bZIP60 fusion protein *in planta* under the control of its own promoter; therefore, it is difficult to compare these two experiments. Our finding is quite intriguing since the unspliced form of bZIP60 mRNA is much more abundant than the spliced form. Iwata et al. [23] detected the endogenous bZIP60 protein in Arabidopsis cell cultures; however, in several organs no clear signal of the protein produced by the unspliced form of bZIP60 could be observed. This situation changed when DTT was used to trigger UPR. Therefore, it is likely that under basal conditions, plants show low or undetectable levels of the protein derived from the unspliced form. On this regard, immunoblot analyses performed in protein extracts obtained from Arabidopsis seedlings, using antibodies against bZIP60 that are able to detect both the spliced and unspliced proteins, showed no evidence for the presence of any protein in basal conditions. However, a predominant band corresponding to bZIP60s, appeared upon treatment with Tm [16]. Interestingly, the use of an inhibitor of the proteasome allowed us to detect the protein in the absence of any UPR-inducer suggesting that in basal conditions, bZIP60u is being degraded by the proteosome. This kind of regulation by the proteasome has been described for the unspliced form of XBP1 (XBP1u), the mammalian ortholog of bZIP60 [35]. In addition, the analysis of the subcellular localization of GFP-bZIP60 in the presence of MG132 under basal conditions indicated that the protein was present in a compartment that co-localizes with an ER-tracker, indicating that bZIP60u is located in this organelle, as it has been previously suggested [23]. Furthermore, no nuclear localization of the protein was observed when no UPR-inducers were applied. Since, no splicing of bZIP60 mRNA was observed when the seedlings were treated with MG132, we concluded that the protein observed correspond to bZIP60u. The incubation with DTT and MG132 led to the splicing of bZIP60 mRNA. This correlates with the accumulation of the signal derived from GFP-bZIP60 in the nucleus. Additionally, a protein was detected by immunoblot analyses. This protein has similar MW as the protein observed during the treatment with MG132 alone. We believe that the protein observed under MG132 treatment could be bZIP60u and the protein present upon treatment with DTT could be bZIP60s. Since the difference is only 37 aminoacid, the change in MW should be around 4 kDa, a difference that is challenging to differentiate in a fusion protein whose MW is around 70 kDa. In addition, we do not know whether some postranslational modifications may affect the electrophoretic mobility making it difficult to distinguish the theoretical differences in MW between the bZIP60s and bZIP60u.

Our results show that splicing of the mRNA that encodes for the transcription factor involved in the IRE1 signalling pathway in plants, the most conserved branch of the unfolded protein response, has several characteristics that are similar to other eukaryotes; however, there are some features that are unique to plants and their physiological importance remains to be determined.

## Supporting Information

S1 Fig A PCR artifact is detected during the amplification of the double hairpin region of bZIP60. (A) CE-LIF analysis of RT-PCR products obtained from total RNA extracted from *ire1a ire1b* mutant Arabidopsis seedlings (7-days-old) treated with DTT (2 mM) or tunicamycin (Tm; 5 μg/mL); and PCR products obtained from an Arabidopsis genomic DNA sample (gDNA). DMSO and water (H2O) treated samples served as mock control for the chemicals. Spherograms peaks corresponding to the unspliced form of bZIP60 (U) are depicted. Black arrows indicate the presence of a small peak in all samples that show an electrophoretic migration similar to the bZIP60 processed form. (B) Amplification of the small peak obtained from gDNA sample. Data are representative of three independent experiments.
(TIF)

S2 Fig DTT and tunicamycin maintain their biological effect after 5 hours of plant treatment. CE-LIF analysis of RT-PCR products obtained from total RNA extracted from wild type Arabidopsis seedlings (7-days-old), treated with DTT (2 mM) or tunicamycin (Tm; 5 μg/mL) for two hours using culture media previously used in wild type Arabidopsis seedlings for 5 hours. All quantification calculations were performed as described in materials and methods section. Data are representative of three independent experiments. Data are shown as mean ± SD.
(EPS)

S3 Fig *bzip17* mutant plants show an altered processing of bZIP60 under salicylic acid treatment. CE-LIF analysis of RT-PCR products obtained from total RNA extracted from wild type (WT), *bzip28* mutant or *bzip17* mutant Arabidopsis seedlings (7-days-old) treated with DTT (2 mM), tunicamycin (Tm; 5 μg/mL), salicylic acid (SA; 0.5 mM) or exposed to high temperature (Heat; 42°C) during two hours. All quantification calculations were performed as described in materials and methods section. Data are representative of three independent experiments. Data are shown as mean ± SD.
(EPS)

S4 Fig The unspliced form of bZIP60 can be detected in the *bzip60*/60pro:GFP-bZIP60 transgenic line under basal conditions. CE-LIF analysis of RT-PCR products obtained from total RNA extracted from wild type (WT) or *bzip60*/60pro:GFP-bZIP60 transgenic Arabidopsis seedlings (7-days-old) under basal conditions. Data are representative of three independent experiments.
(TIF)
