# Supplementary figures and images for: The Dynamic of the Splicing of bZIP60 and the Proteins Encoded by the Spliced and Unspliced mRNAs Reveals Some Unique Features during the Activation of UPR in Arabidopsis thaliana
Source: PLoS One. 2015 Apr 10;10(4):e0122936. doi: 10.1371/journal.pone.0122936 (PMC4393087; doi:10.1371/journal.pone.0122936)

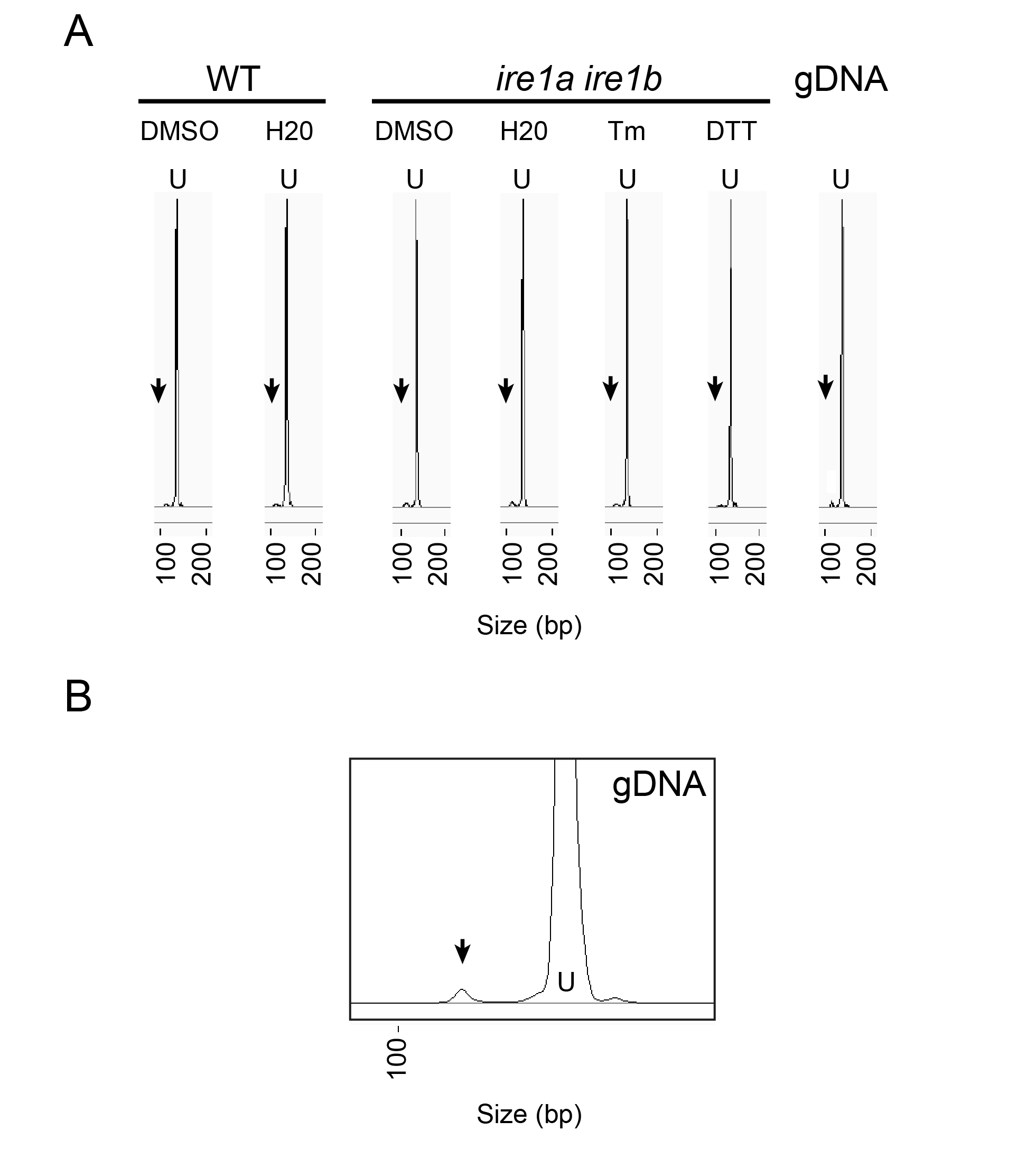

Supplement: S1 Fig — (A) CE-LIF analysis of RT-PCR products obtained from total RNA extracted from ire1a ire1b mutant Arabidopsis seedlings (7-days-old) treated with DTT (2 mM) or tunicamycin (Tm; 5 μg/mL); and PCR products obtained from an Arabidopsis genomic DNA sample (gDNA). DMSO and water (H2O) treated samples served as mock control for the chemicals. Spherograms peaks corresponding to the unspliced form of bZIP60 (U) are depicted. Black arrows indicate the presence of a small peak in all samples that show an electrophoretic migration similar to the bZIP60 processed form. (B) Amplification of the small peak obtained from gDNA sample. Data are representative of three independent experiments. (TIF) [file pone.0122936.s001.tif]

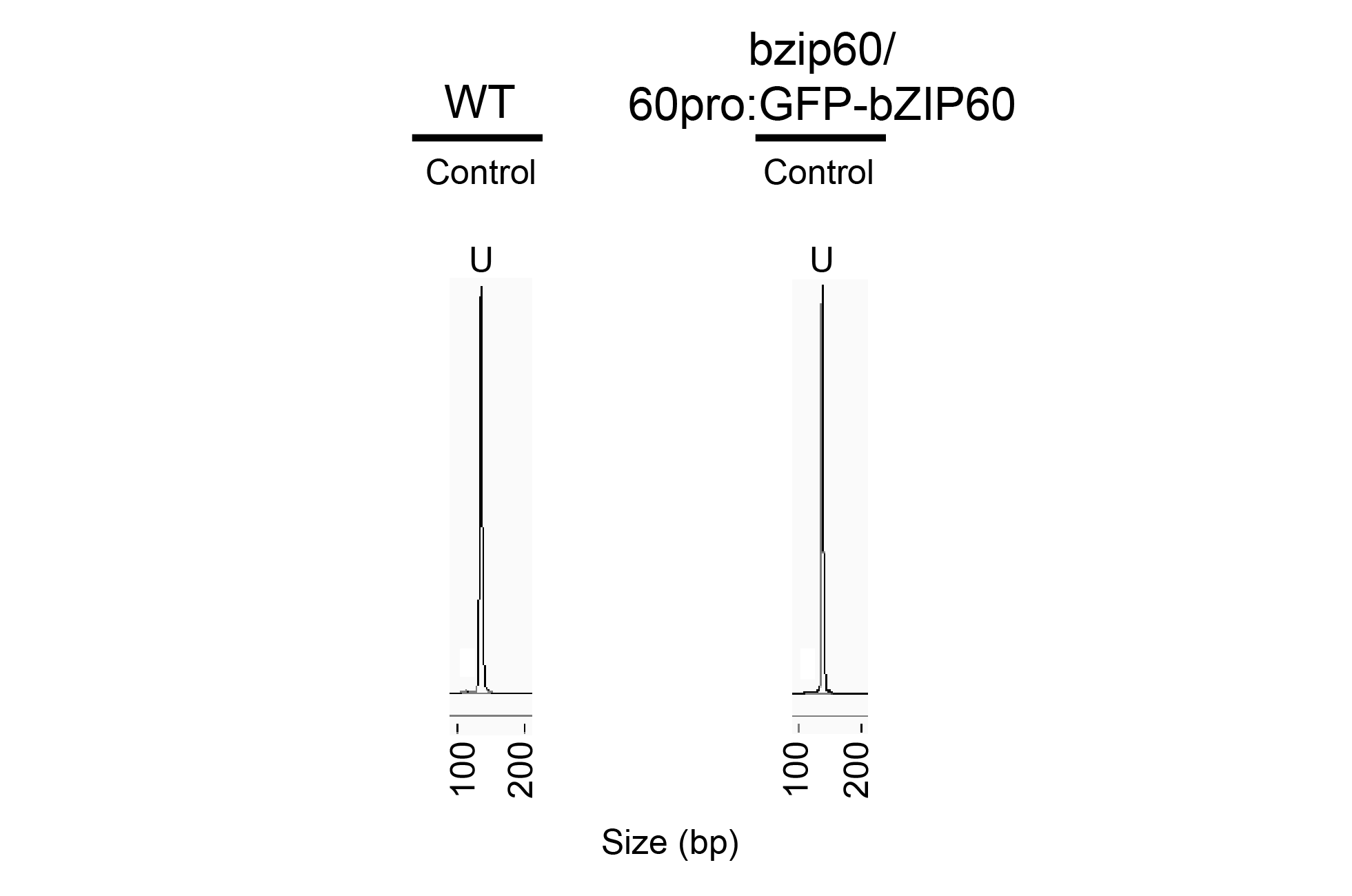

Supplement: S4 Fig — CE-LIF analysis of RT-PCR products obtained from total RNA extracted from wild type (WT) or bzip60/60pro:GFP-bZIP60 transgenic Arabidopsis seedlings (7-days-old) under basal conditions. Data are representative of three independent experiments. (TIF) [file pone.0122936.s004.tif]
